# Severe Atypical Optic Neuritis in a Patient With Pemphigus Foliaceus on Immunosuppressive Therapy

**DOI:** 10.7759/cureus.16010

**Published:** 2021-06-29

**Authors:** YVK Chaitanya, Ashok Kumar, Jaya Kaushik, Aanchal Singhal, Srujana Dubbaka

**Affiliations:** 1 Ophthalmology, Armed Forces Medical College, Pune, IND

**Keywords:** atypical, bilateral optic neuritis, pemphigus foliaceous, immunotherapy, steroids

## Abstract

Bilateral simultaneous acute optic neuritis is a rare occurrence in adults, which has been reported mostly in the setting of untreated systemic autoimmune disorders. Such clinical presentations are encompassed in the spectrum of ‘atypical’ optic neuritis with resultant poor visual outcome and mainly associated with neuromyelitis optica spectrum disorders. We report an unusual presentation, that is, severe atypical optic neuritis in a patient of pemphigus foliaceous on immunosuppressive therapy with significant visual recovery after treatment.

## Introduction

Optic neuritis is a condition that is characterized by inflammation of the optic nerve typically presenting unilaterally in young adults with a preponderance in females and frequently associated with multiple sclerosis. Bilateral simultaneous acute optic neuritis is a rare occurrence in adults, which has been reported mostly in the setting of systemic autoimmune disorders and is encompassed in the spectrum of ‘atypical’ optic neuritis. In atypical optic neuritis, the inflammation may be triggered by an inflammatory or immune-related disease like sarcoidosis, neuromyelitis optica (NMO), or by a different process such as infection (e.g., Lyme disease, syphilis) or vitamin B12 deficiency.

Autoimmune optic neuropathy has been described in the presence of serological evidence of an autoimmune disorder and must be considered when a patient with optic neuritis has atypical features. Pemphigus foliaceus is an acquired autoimmune disorder characterized by blistering and intra-epidermal deposition of IgG auto-antibodies against intracellular adhesion glycoprotein desmoglein-1. An isolated case of NMO in a patient with pemphigus foliaceus has been reported till date.

We report a case of bilateral severe atypical optic neuritis with profound visual loss at presentation in a patient of pemphigus foliaceus on immunosuppressive therapy, where NMO was ruled out and a favorable outcome was observed following high-dose systemic corticosteroid therapy.

## Case presentation

**Figure 1:**
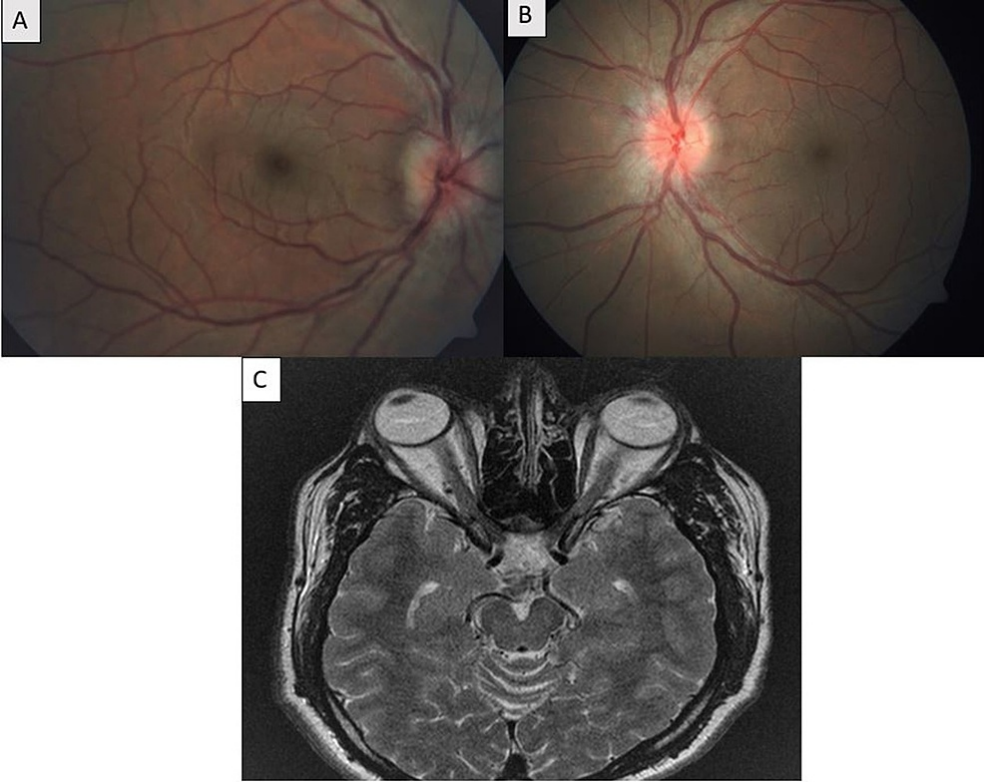
Pre-treatment fundus and MR images of the patient. (A, B) Fundus imaging photos of right and left eyes showing bilateral disc edema, inferior disc hemorrhages, and peripapillary retinal nerve fiber layer edema suggestive of severe papillitis.  (C) MRI of brain and orbits revealing bilateral optic nerve thickening in the intra-orbital portion consistent with optic neuritis and absence of any demyelinating lesions in brain.

**Figure 2:**
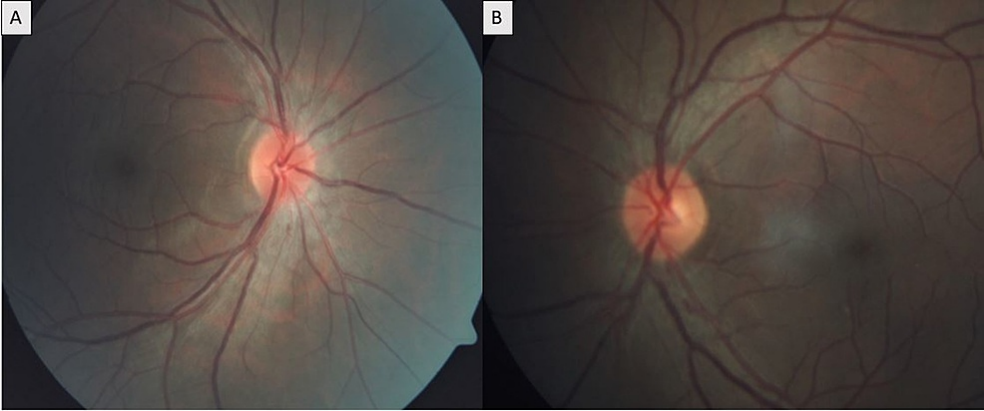
Post-treatment fundus images of the patient. (A, B) Fundus imaging photos of right and left eyes at 10 days after treatment showing complete resolution of bilateral disc edema and clearance of peripapillary hemorrhages.

A 37-year-old male presented to our outpatient department with complaints of rapid, progressive profound vision loss over last three days. He was a known case of pemphigus foliaceus on immunomodulators for last three years, but was irregular with follow-up and non-compliant to medications. On ocular evaluation, his best-corrected visual acuity was absence of light perception in both eyes with bilateral afferent pathway defect, amaurotic pupil, and painful ocular movements at extremes of gazes. Fundus evaluation revealed bilateral disc edema, inferior disc hemorrhages, and peripapillary retinal nerve fiber layer edema suggestive of severe papillitis (Figure 1A, 1B). An urgent MRI brain, orbits, and whole spine was done, which suggested bilateral optic nerve thickening in the intra-orbital portion consistent with optic neuritis and no demyelinating lesions in brain or spinal cord (Figure 1C). Fundus fluorescein angiography showed normal filling time and pattern of all the retinal vessels. Visual evoked potential test showed absent response bilaterally. He was diagnosed as a case of atypical optic neuritis and was started on intravenous methylprednisolone 1 g/day under cardiac monitoring on the same day for five days followed by oral prednisolone for 11 days tapered over three days and concurrently investigated for other causes of optic neuritis. All investigations including anti-MOG antibody (Ab) and anti-AQP4 Ab were normal. Cerebrospinal fluid (CSF) analysis for cells, protein, and sugar levels, oligoclonal bands/immunoglobulins, and staining was normal. Over a course of 10 days, the patient reported significant improvement in vision to 5/60 in both eyes and fundus examination revealed resolution of bilateral disc edema and disappearance of peripapillary hemorrhage (Figure 2A, 2B). He was subsequently discharged on tapering doses of oral prednisolone and immunosuppressive therapy in the form of azathioprine (1.5 mg/kg).

## Discussion

Atypical optic neuritis is usually seen as an early manifestation of NMO or NMO spectrum disorder (NMOSD) [1,2]. Atypical findings of acute demyelinating optic neuritis include male gender, profound diminution of vision with no or minimal recovery, optic disc and or retinal hemorrhages, severe optic disc swelling, macular exudates, and absence of pain or pain persisting for more than a few days [3]. Patients with NMO have severe optic neuritis with a slow recovery, which can be bilateral and chronic, often leading to blindness.

Our present case is a middle-aged male, a known case of pemphigus foliaceus on irregular immunosuppressive therapy who developed atypical optic neuritis after a few years of treatment. The most probable cause of such presentation, NMO or NMOSD, was ruled out with relevant investigations. The patient also had multiple atypical findings like male gender, bilateral non-perception of light, severe papillitis, and optic disc hemorrhages. But, it did not correlate with any common cause of atypical optic neuritis as suggested by the investigations for immunological and/or infective disorders as well as CSF analysis for cells, protein, or sugar levels as well as immunoglobulins. Atypical optic neuritis can have devastating effects on the vision of the patient if not treated timely, and even then, the results are less than satisfactory in a significant number of cases.

A single case of NMO-associated optic neuritis in a patient with pemphigus foliaceus has been reported till date [4]. Velez et al. reported that patients affected by a type of endemic pemphigus foliaceus exhibit autoantibodies to optic nerve sheath envelope cell junctions, and these seem to alter vision in the patients [5]. We also hypothesize that irregular immunosuppression in the present case could have precipitated the optic neuritis. Thus, our case is the first report of severe atypical optic neuritis seen in a patient with pemphigus foliaceus despite being on immunosuppressive therapy where timely diagnosis and aggressive management resulted in a significant improvement in visual acuity of the patient.

## Conclusions

Therefore, it is critical to recognize and monitor patients with autoimmune disorders for possible development of atypical optic neuritis and institute prompt management, so as to achieve better visual outcomes and reduce ocular morbidity. 
